# Electrorheology of SI-ATRP-modified graphene oxide particles with poly(butyl methacrylate): effect of reduction and compatibility with silicone oil

**DOI:** 10.1039/c8ra08518h

**Published:** 2019-01-09

**Authors:** Miroslav Mrlik, Marketa Ilcikova, Josef Osicka, Erika Kutalkova, Antonin Minarik, Alenka Vesel, Jaroslav Mosnacek

**Affiliations:** Centre of Polymer Systems, University Institute, Tomas Bata University in Zlin Trida T. Bati 5678 760 01 Zlin Czech Republic mrlik@utb.cz marketa.ilcikova@savba.sk; Polymer Institute, Slovak Academy of Sciences Dúbravska cesta 9 845 41 Bratislava 45 Slovakia; Department of Physics and Materials Engineering, Faculty of Technology, Tomas Bata University in Zlin Vavrečkova 275 760 01 Zlin Czech Republic; Jozef Stefan Institute Jamova 39 1000 Ljubljana Slovenia; Department of Polymer Engineering, Faculty of Technology, Tomas Bata University in Zlin Vavreckova 275 CZ-76272 Zlin Czech Republic

## Abstract

Surface-initiated atom transfer radical polymerization (SI-ATRP) was used to modify graphene oxide (GO) particles with poly(butyl methacrylate) (PBMA) chains. This procedure facilitated the single-step fabrication of a hybrid material with tailored conductivity for the preparation of a suspension in silicone oil with enhanced sedimentation stability and improved electrorheological (ER) activity. PBMA was characterized using various techniques, such as gel permeation chromatography (GPC) and ^1^H NMR spectroscopy. Thermogravimetric analysis through on-line investigation of the Fourier transform infrared spectra, together with transmission electron microscopy, X-ray photoelectron microscopy, and atomic force microscopy, were successfully used to confirm GO surface modification. The ER performance was investigated using optical microscopy images and steady shear rheometry, and the mechanism of the internal chain-like structure formation was elucidated. The dielectric properties confirmed enhanced ER performance owing to an increase in relaxation strength to 1.36 and decrease in relaxation time to 5 × 10^−3^ s. The compatibility between GO and silicone oil was significantly influenced by covalently bonded PBMA polymer brushes on the GO surface, showing enhanced compatibility with silicone oil, which resulted in the considerably improved sedimentation stability. Furthermore, a controlled degree of reduction of the GO surface ensured that the suspension had improved ER properties.

## Abstract 2

Surface-initiated atom transfer radical polymerization (SI-ATRP) was used to modify graphene oxide (GO) particles with poly(butyl methacrylate) (PBMA) chains.

## Graphical abstract

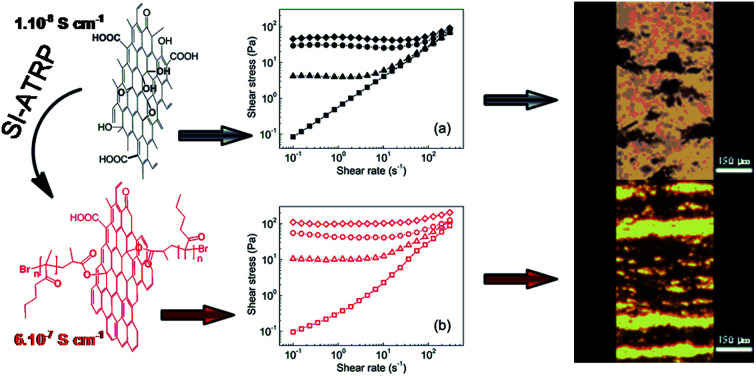

## Introduction

Electroresponsive systems, also known as Smart Systems, are groups of materials whose physical properties can be varied by applying an external electric field.^1^ Electrorheological (ER) suspensions are a type of electroresponsive system,^2^ which essentially comprise semiconducting particles that can be induced with dipoles upon application of an electric field and insulating fluid.^3^ Particles homogenously dispersed in the medium can create internal chain-like structures upon application of an external electric field. Such structure formation results in a change in viscosity of several orders of magnitude.^4^ This process is completely reversible, with the system reverting to its initial state after the electric field is switched off. This phenomenon has found various industrial applications, such as in damping,^5^ haptic displays,^6^ and invasive surgery.^7^

In the last two decades, various materials, mainly based on inorganic particles (titanium oxide^8^ or silica^9,10^), conducting polymers (poly(aniline),^11^ poly(pyrrole),^12^ or poly(diphenyl amine),^13^) and their core–shell analogues^14,15^ and hybrids,^16^ have been used as the dispersed phase in ER suspensions. Recently, Choi *et al.*^17^ found that graphene oxide (GO) is a promising material for application in ER suspensions. Therefore, various researchers have extensively studied neat GO^18^ and its core–shell particles^19,20^ or hybrids,^21,22^ and their effect on ER performance. Slight synergism has been obtained for GO core–shell particles, in which conducting polymers^23^ or polar modifying agents play important roles,^24^ owing to their tunable conductivity.

The surface of particles and nanoparticles can be modified by polymer chains through either reactions of polymer functional groups with the functional groups present on the particle surface^25,26^ or direct polymerization, where “grafting from” or “grafting through” methods can be applied.^27^ Various controlled/living polymerizations (CLP) allow “grafting from” modifications through surface-initiated polymerization, achieving polymer synthesis with controlled molar mass, narrow dispersity, and various functionalities.^28^ Atom transfer radical polymerization (ATRP) is currently the most widely used reversible deactivation radical polymerization technique owing to the availability of transition metal catalysts and large-scale monomers, which can be polymerized using this technique.^29^ Furthermore, ATRP initiator can easily be attached to various surfaces for subsequent surface-initiated ATRP (SI-ATRP). We recently reported that GO can be grafted with polystyrene chains using SI-ATRP while simultaneously reducing the GO particles in the presence of tertiary amines commonly used as ligands in ATRP.^30^ The GO reduction ability during SI-ATRP modification was an additional advantage of this ATRP technique. This approach was also confirmed to be valid for poly(glycidyl methacrylate), with the final partially reduced and grafted GO improving the ER performance of GO-based suspensions.^22^ Owing to these promising results, other monomers, such as butyl methacrylate, were selected for GO grafting in this study to further improve compatibility with silicon oil through the aliphatic chain present in the monomer structure, and because various inorganic/polymer hybrids based on materials with stronger dipole moments exhibit a synergistic effect on ER performance.^31–34^

## Experimental

### Materials

Graphite (powder, <20 μm, synthetic) was used as a precursor for GO sheets. Sulfuric acid (H_2_SO_4_, reagent grade, 95–98%), sodium nitrate (NaNO_3_, ACS reagent, ≥99%), potassium permanganate (KMnO_4_, 97%), and hydrogen peroxide (H_2_O_2_, ACS reagent, 29.0–32.0 wt% H_2_O_2_ basis) were used as chemical reagents to obtain suitable exfoliation conditions for forming GO sheets. α-Bromoisobutyryl bromide (BiBB, 98%) served as an initiator linked to the GO surface. Initiator bonding was performed in the presence of proton scavenger triethylamine (TEA, ≥99%). Butyl methacrylate (BMA, 99%), ethyl α-bromoisobutyrate (EBiB, 98%), *N*,*N*,*N*′,*N*′′,*N*′′-pentamethyldiethylenetriamine (PMDETA, ≥99%), copper bromide (CuBr, ≥99%), and anisole (99%) were used as a monomer, initiator, ligand, catalyst, and solvent, respectively. Diethyl ether (ACS reagent, anhydrous, ≥99%) was used as a drying agent. All chemicals were purchased from Sigma Aldrich (USA) and used without further purification (except BMA). BMA was purified by passing through a neutral alumina column to remove *p-tert*-butylcatechol as inhibitor prior to use. Hydrochloric acid (HCl, 35%, p.a.), ethanol (absolute anhydrous, p.a.), tetrahydrofuran (THF, p.a.), toluene (p.a.), and acetone (p.a.) were obtained from Penta Labs (Czech Republic). Deionized (DI) water was used in corresponding experimental processes and washing routines. Lukosiol M200 grade is silicone oil with a viscosity of 200 mPa s, as supplied by Lukosiol (Kolín, Czech Republic).

### Preparation of GO modified with poly(butyl methacrylate) (GO-PBMA)

GO particles were synthesized from graphite powder using a modified Hummers method,^35^ as described in our previous study.^36^ Hydroxyl groups on the GO surface were reacted with BiBB to covalently bond the ATRP initiator to the surface using the following procedure: GO (2 g), dried THF (60 mL), and TEA (12 mL) were mixed under an argon atmosphere at approximately 5 °C (ice/water bath), and BiBB (7 mL) was added dropwise for 1 h, followed by further mixing at ambient temperature overnight. The reaction mixture was consecutively washed with THF (150 mL), acetone (150 mL), and finally water (2 × 200 mL). Modified GO was then filtered off using a PTFE filter (pore size, 0.44 μm). Excess water was removed from the treated particles by washing with diethyl ether (3 × 60 mL).

PBMA was grafted from the GO surface using the SI-ATRP approach, as follows: GO containing bonded ATRP initiator (0.5 g) was placed into a Schlenk flask equipped with a gas inlet/outlet and septum. The flask was evacuated and backfilled with argon three times. The argon-purged chemicals, namely BMA (15.4 mL, 130.5 mmol), EBiB (0.192 mL, 1.305 mmol), PMDETA (1.090 mL, 5.220 mmol), and anisole (15 mL), were gradually added. The presence of oxygen was further minimized by degassing the system using several freeze–pump–thaw cycles. In a frozen state, the CuBr catalyst (187.2 mg, 1.305 mmol) was added under gentle argon flow and the flask was immersed into a silicone oil bath pre-heated to 60 °C for 2 h to achieve polymerization. The molar ratio of the reactants (BMA/EBiB/CuBr/PMDETA) was 100 : 1:1 : 4, while anisole (50 vol%) was used as solvent. During polymerization, the viscosity of the mixture gradually increased. The reaction was stopped by exposing the mixture to air and cooling to ambient temperature. The product was purified by filtration, washing with DMF (3 × 100 mL) and diethyl ether (3 × 50 mL). The final product was dried in desiccator under ambient conditions.

### Characterization techniques

The molar mass and dispersity of the PBMA chains grown from the sacrificial initiator were determined by GPC using a PL-GPC220 instrument (Agilent, Japan) with THF as solvent at a flow rate of 1.0 mL min^−1^. Polystyrene was used as a standard and anisole as an internal standard. Monomer conversions were determined by ^1^H NMR using a 400 MHz VNMRS Varian NMR spectrometer equipped with a 5 mm 1H–19F/15N–31P PFG AutoX DB NB probe at 25 °C and using deuterated chloroform as solvent. Infrared spectra of the fillers were recorded on a Nicolet 6700 spectrometer (Thermo Scientific, USA) equipped with a SMART ATR accessory with Ge crystal. Raman spectra were measured on a Nicolet DXR spectrometer (Nicolet, USA) using an excitation wavelength of 532 nm (3 scans, resolution of 2 cm^−1^). The integration time was 30 s, while the laser power on the surface was set to 1 mW. Oxygen-containing groups present on the GO surface were investigated by thermogravimetric analysis (TGA/SDTA 851e, Mettler Toledo, Switzerland) using a heating rate of 10 K min^−1^ under a nitrogen atmosphere. Transmission electron microscopy (TEM) images were acquired with resolution of 0.35 nm using a Philips CM12 instrument (Philips, Amsterdam, Netherlands).

Conductivity was measured using the van der Pauw method (Keithley 6517B, USA) at room temperature. Powders of the synthesized neat GO and modified GO-PBMA samples were pressed into pellets (diameter, 13 mm; thickness, 0.3–0.4 mm) at 400 MPa. The density of the GO-based pellets was determined by weighing before and after immersion in *n*-decane using a Sartorius R160P analytical balance (Sartorius AG, Germany).

XPS measurements were performed using a TFA XPS device from Physical Electronics. The base pressure in the XPS analysis chamber was approximately 6 × 10^−8^ Pa. The samples were excited over a spot area of 400 μm^2^ using monochromatic Al Kα_1,2_ radiation at 1486.6 eV. Photoelectrons were detected with a hemispherical analyser positioned at a 45° angle with respect to the surface normal of the sample. The energy resolution was approximately 0.5 eV. Survey-scan spectra were acquired at a pass energy of 187.85 eV, while for C1s, individual high-resolution spectra were recorded at a pass energy of 29.35 eV and with an energy step of 0.125 eV. All spectra were referenced to the main C1s peak of the carbon atoms, which was assigned a value of 284.8 eV. The spectra were analysed using MultiPak v8.1c software (Ulvac-Phi Inc., Kanagawa, Japan, 2006) from Physical Electronics (supplied with the spectrometer). C1s spectra were fitted with a symmetrical Gauss–Lorentz function. A Shirley-type background subtraction was used.

Dried GO sheets on a mica surface were characterized using atomic force microscopy (AFM; Dimension Icon atomic force microscope, Bruker). Measurements were performed at a scan speed of 1 Hz with a resolution of 512 × 512 pixels in ScanAsyst mode at room temperature under an air atmosphere. A ScanAsyst-Air probe with a resonant frequency of 70 kHz and a stiffness constant of 0.4 N m^−1^ (Bruker) was used. AFM data were processed using Gwyddion 2.51 software (Czech Metrology Institute).

Suspensions (5 wt% of dispersed phase) were mixed according to the following procedure: for all measurements, GO particles or GO-PBMA analogues were dispersed in silicone oil by first agitating mechanically for 5 min and then ultrasonicating for 30 s. Rheological measurements were performed in controlled shear rate (CSR) mode using a rotational viscometer (Bohlin Gemini, Malvern Instruments, UK). The suspensions were placed into parallel-plate geometry (diameter, 40 mm; gap, 0.5 mm). The electrorheological cell was connected to a high-voltage DC source (TREK 668B, USA) to generate electric field strengths of 0–2.5 kV mm^−1^. Before each measurement, the previously built-up particulate structures were destroyed by shearing the sample at a shear rate of 50 s^−1^ for 60 s. All measurements were performed at 25 °C.

Suspensions containing 1 wt% of GO-based particles were mixed with silicone oil and injected between two copper electrodes (gap, 80 μm) and connected to a high-voltage source (Keithley 2410, USA). Development of the internal chain-like structures was recorded using an optical microscope (N 400M, China).

The ER mechanism of internal chain-like structure development was investigated using the power law model shown in eqn (1).^4^

1 *τ*_y_ = *qE*^*α*^

where *τ*_y_ is the yield stress, *E* is the electric field strength, *α* is the particle response to electric field application, and *q* is related to the internal structure stiffness.

The dielectric properties were measured by impedance dielectric spectroscopy using a Novocontrol Concept 50 analyzer (Novocontrol, Germany) connected to cylindrical sample cell BDS 1307 for liquid materials. Dielectric properties, such as the relative permittivity (*ε*′) and dielectric loss factor (*ε*′′), were investigated in the frequency range of 0.5 Hz to 2 MHz. Dielectric spectra were analysed using the Havriliak–Negami model (eqn (2)).^37^2
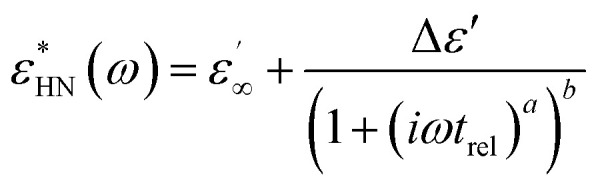
where 
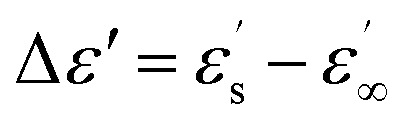
 is the dielectric relaxation strength, 
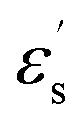
 and 
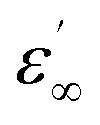
 are the relative permittivities at zero and infinite frequencies (*f*), respectively, *ω* is the angular frequency (2π*f*), *t*_rel_ is the relaxation time, and *a* and *b* are shape parameters describing the asymmetry of the dielectric function.

## Results and discussion

### Modification of GO with poly(butyl methacrylate)

The ATRP conditions for GO surface modification were set to obtain sufficiently long PBMA chains to improve the compatibility of the GO particles with silicone oil. This improved the stability of the final suspension in a short polymerization time, during which slight reduction of the GO surface was also obtained to afford conductivity suitable for magnetorheological suspensions. Therefore, polymerization for 2 h, the BMA conversion was approximately 41%, while the molar mass and dispersity of the PBMA chains were 5920 g mol^−1^ and 1.23, respectively. The molar mass fitted quite well with the theoretical mass and the dispersity was narrow, showing that ATRP of BMA under the applied conditions was well controlled. Furthermore, to carefully characterize the GO-PBMA hybrid particles, the grafting density was calculated. There are various approaches to calculating the grafting density, such as simple calculation from TGA, as used by Zhao *et al.* and Zhang *et al.*^38,39^ However, in this case, a more precise calculation was used, involving TGA data, the molar mass of the polymer grafts, and the shape parameters of the particles, as previously reported.^40,41^ The GO particle density (*ρ*) of 2.54 g cm^3^, GO sheet thickness from AFM (*l*) of 2 nm, and calculated specific surface area (S) of 400 m^2^ g^−1^ were successfully implemented. The grafting density was then calculated to be 0.01 chain per nm^2^, showing a relatively low density of surface modification.

**Fig. 1:**
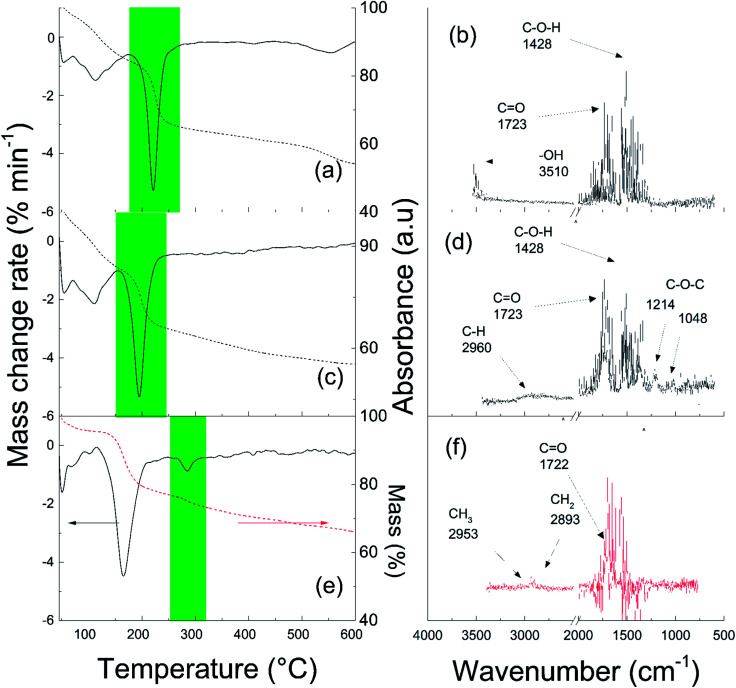
TGA-FTIR spectra of (a and b) neat GO, (c and d) GO with initiator, and (e and f) GO-PBMA.

To confirm the successful modification of the GO particles with PBMA chains, the volatile products formed during TGA analysis were monitored on-line using FTIR. As shown in Fig. 1, the decomposition of oxygen-containing groups on the surface of GO was observed. The major portion of such substances were cleaved in the temperature range of 180–260 °C (Fig. 1a), as reflected in the C–OH absorption band at 1428 cm^−1^, C=O band at 1723 cm^−1^, and –OH resonance at 3510 cm^−1^ in the FTIR spectra (Fig. 1b). For sample GO-I, decomposition was observed in the temperature range of 150–250 °C (Fig. 1c). This was indicated by both the original oxygen-containing groups and ATRP initiator moieties covalently attached to the GO surface, probably in close proximity, reflecting the same absorption bands, but also to additional C–O–C vibrations at 1214 cm^−1^ and 1048 cm^−1^ and a C–H vibration at 2960 cm^−1^ from the initiator (Fig. 1d). For GO-PBMA, an additional peak appeared in the temperature range of 260–315 °C that reflected the decomposition of covalently bonded PBMA (Fig. 1e). The connection of this peak with the C=O absorption band at 1722 cm^−1^ and aliphatic chain C–H vibrations at 2953 cm^−1^ and 2893 cm^−1^ (Fig. 1f) confirmed the presence of PBMA on the GO sheet surface.

**Fig. 2:**
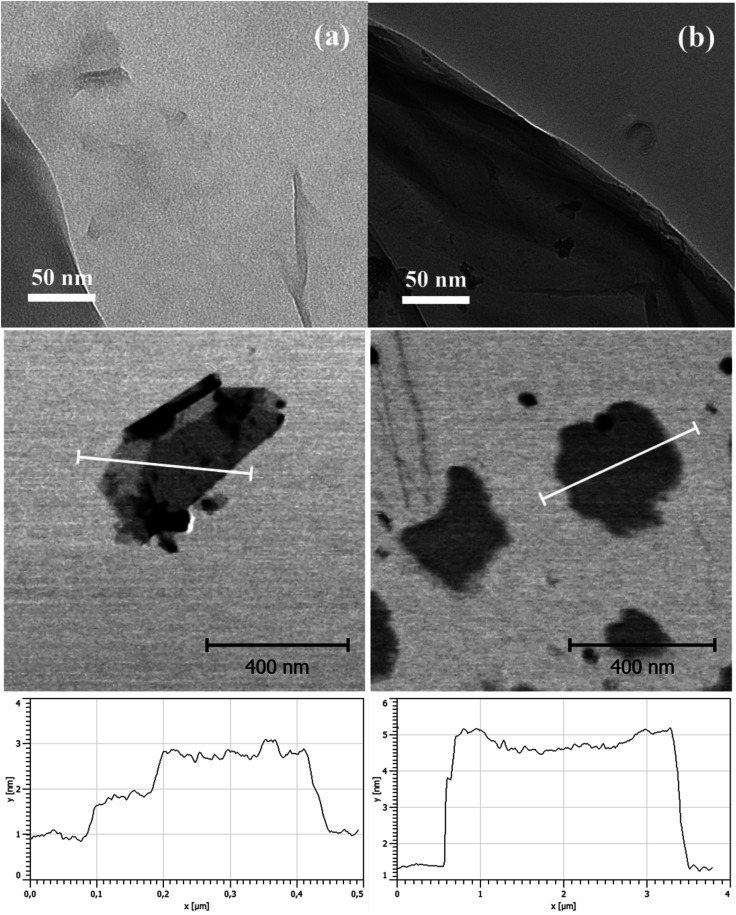
Transmission electron microscopy images, atomic force microscopy images, and thickness profile of (a) neat GO and (b) GO-PBMA.

TEM images were used for visual confirmation of the PBMA layer on the GO surface. As clearly shown in Fig. 2a, neat GO was very well exfoliated and had only up to a few layers in its layered structure. In contrast, GO modification with PBMA was observed as a floss-like layer, which made the GO slightly darker and less sharp at the edges in the final TEM image.

To support the TEM microscopy findings, AFM images of both the neat GO and GO-PBMA particles were acquired (Fig. 2c and d). These clearly showed that neat GO particles consisted of one or two layers (Fig. 2c) with 1 or 2 nm thick sheets along the particle width, respectively, similar to previously reported observations.^3,42^ Darker parts of the figures represented additional particles, mostly at the nanoscale (several nanometres), or sheets perpendicular to the investigated sheet. In contrast, clear confirmation of the presence of a PBMA layer on the particle was observed when significantly more individual GO-PBMA sheets were visible, due to improved dispersibility and significant repulsion between the particles. Furthermore, particle thickness increased from 1 to 5 nm, showing that the polymer layer was around 3 nm thick, which corresponded well with the low grafting density.

**Fig. 3:**
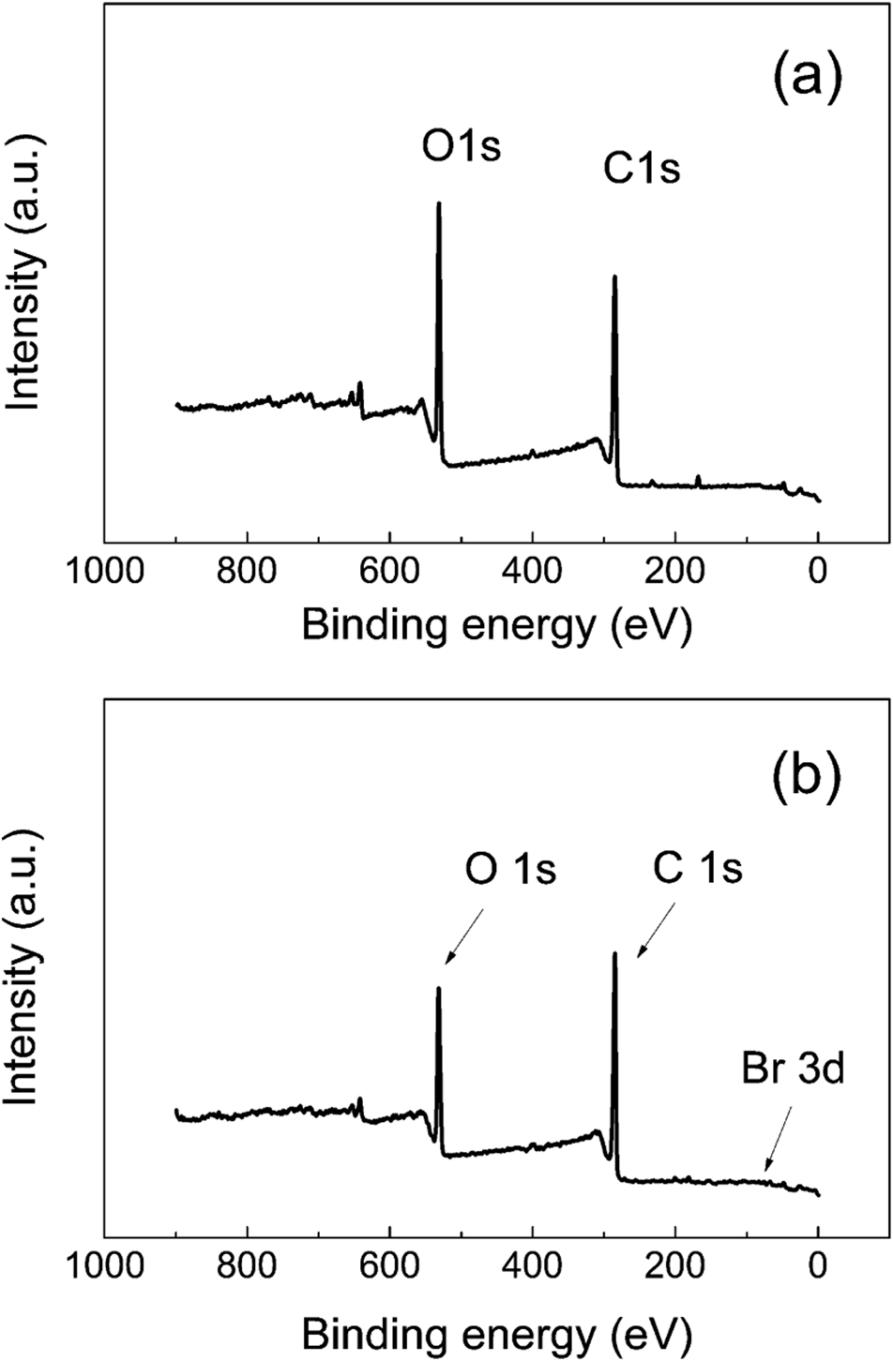
XPS spectra of (a) neat GO and (b) GO-PBMA particles.

The surface properties of neat GO and GO-PBMA were characterized in detail using XPS. For GO surface modification, hydroxyl groups needed to be present on the GO surface to allow covalent bonding with the ATRP initiator for subsequent polymerization. XPS analysis (Table 3) showed that about 32% of carbon was bonded with oxygen through a single bond, which was expected to provide a sufficient amount of reactive sites for attachment of the ATRP initiator. Fig. 3 clearly shows that the amount of oxygen-containing groups decreased after modification with the PBMA polymer shell, while the amount of carbon slightly increased. There were also some visible impurities, calculated as 0.5% in neat GO and 0.3% in GO-PBMA, respectively. GO-PBMA also showed a slightly visible bromine Br3d peak (Fig. 3b) that was part of the coating structure, showing a total content of 0.2%. After deconvolution, 95% of this area belonged to the covalently bonded C–Br (terminating the PBMA graft) as visible peaks at 72.0 eV and 70.5 eV, with only 5% showing free Br^−^ (impurities from catalyst) as peaks at 68.9 eV and 67.6 eV. These results correlated well with those observed for brominated graphene.^43^ Furthermore, the spectra intensities shown in Fig. 3b are lower due to substantial coating, similar to that reported by Li *et al.*^3^ Further characterization of the GO and GO-PBMA surfaces was performed by C1s peak deconvolution, which identified individual oxygen–carbon groups, as summarized in Table 1. In accordance with the low grafting density, only a slight increase in the C1s/O1s ratio was observed. Furthermore, after SI-ATRP, an increase in the C1s sp^2^ content was also observed resulting from partial reduction of the GO surface. GO reduction was also investigated using Raman spectroscopy and conductivity measurements, as presented below. Reduction of the GO surface during SI-ATRP can be expected to lead to partial cleavage of the grafted polymer chains, providing a lower grafting density, which is typical for surface-initiated polymerizations. To prove this hypothesis, more detailed study is needed, which is outside the scope of the present study.

**Table 1:** Surface chemical composition of neat GO, GO-I, and GO-PBMA particles. All numbers are in atom%

Sample name	C1s	O1s	C1s sp^2^	C1s sp^3^	C1s C–O	C1s C=O	C1s O–C=O	C1s/O1s
GO	66.7	33.3	26.7	28.4	32.3	9.0	3.6	2.00
GO-I	67.2	32.8	27.9	28.2	31.9	8.6	3.4	2.05
GO-PBMA	70.9	29.1	36.5	25.3	27.5	7.7	3.0	2.43

### Reduction of GO during SI-ATRP

**Fig. 4:**
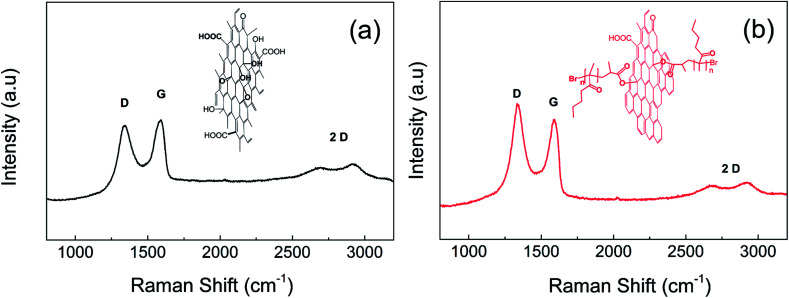
Raman shift of (a) neat GO and (b) GO-PBMA particles.

The reduction of GO particles plays an important role in their further application as a dispersed phase in ER suspensions because the values of electrical conductivity for neat GO are not sufficient for this type of application, while a slightly enhancement of conductivity by 2–3 orders of magnitude should provide a system with improved ER efficiency.^44^ As mentioned above, the tertiary amine used as a ligand in ATRP can also act as a GO reducing agent.^30^ Therefore, conductivity and Raman shift measurements of the GO pellets were performed to confirm the reduction of GO during the SI-ATRP of BMA. Although the conductivity of neat GO was approximately of 1 × 10^−8^ S cm^−1^, the conductivity of GO after the SI-ATRP of BMA was increased to 6 × 10^−7^ S cm^−1^. The reduction of GO was also confirmed by calculating the peak intensities of D to G, which reflected the sp^2^ and sp^3^ hybridized forms of GO, using Raman spectroscopy (Fig. 4). This showed that the recoverability of the conducting pathways on the GO surface was similar to those reported elsewhere.^30,45^*I*_d_/*I*_g_ was calculated to change from 0.90 for neat GO to 1.09 for GO-PBMA. Furthermore, the 2D structure sustained after modification indicated that retained layer was very thin, in good agreement with the TEM and AFM observations. Therefore, the density of the GO nanoparticles also changed only slightly from 2.54 g cm^−3^ for neat GO to 2.21 g cm^−3^ for GO-PBMA. These results clearly showed that the origin of electrical conductivity enhancement was based on GO reduction rather than other phenomena, such as electronic interactions, because even the polymer layer showed pronounced repulsion between individual sheet-like GO particles.

### Optical microscopy of chain-like internal structures

**Fig. 5:**
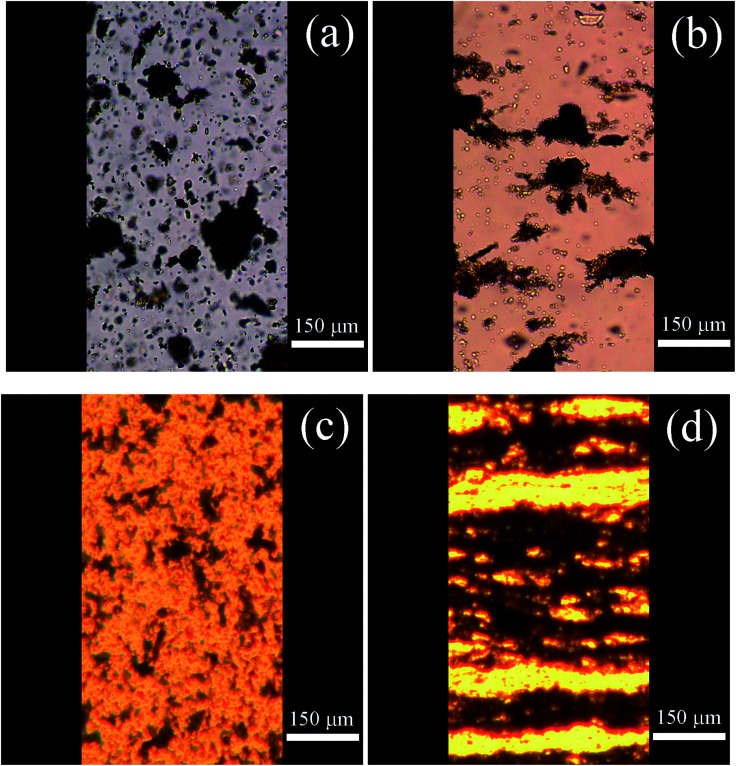
Optical microscopy images of (a and b) neat GO and (c and d) GO-PBMA dispersed in silicone oil in the (a and c) absence and (b and d) presence of an external electric field strength of 0.5 kV mm^−1^.

Optical microscopy is a useful tool for investigating chain-like structures formed in the presence of an external electric field. As shown in Fig. 5a, oxygen-containing groups present on the surface of neat GO particles could not ensure their sufficient dispersion in the silicone oil, resulting in marked GO aggregation. Furthermore, owing to low conductivity, the system based on neat GO was not able to develop proper internal structures after applying an electric field (Fig. 5b), resulting in only weak ER performance, as discussed in the next section. In contrast, GO-PBMA particles were well dispersed in the silicone oil and did not form aggregates owing to the substantial poly(butyl methacrylate) layer on the surface of the GO particles (Fig. 5c). Furthermore, such particles enabled a fast response to application of an electric field and created well-developed chain-like structures (Fig. 5d) essential for good ER performance.

### Steady shear investigations under an external electric field

**Fig. 6:**
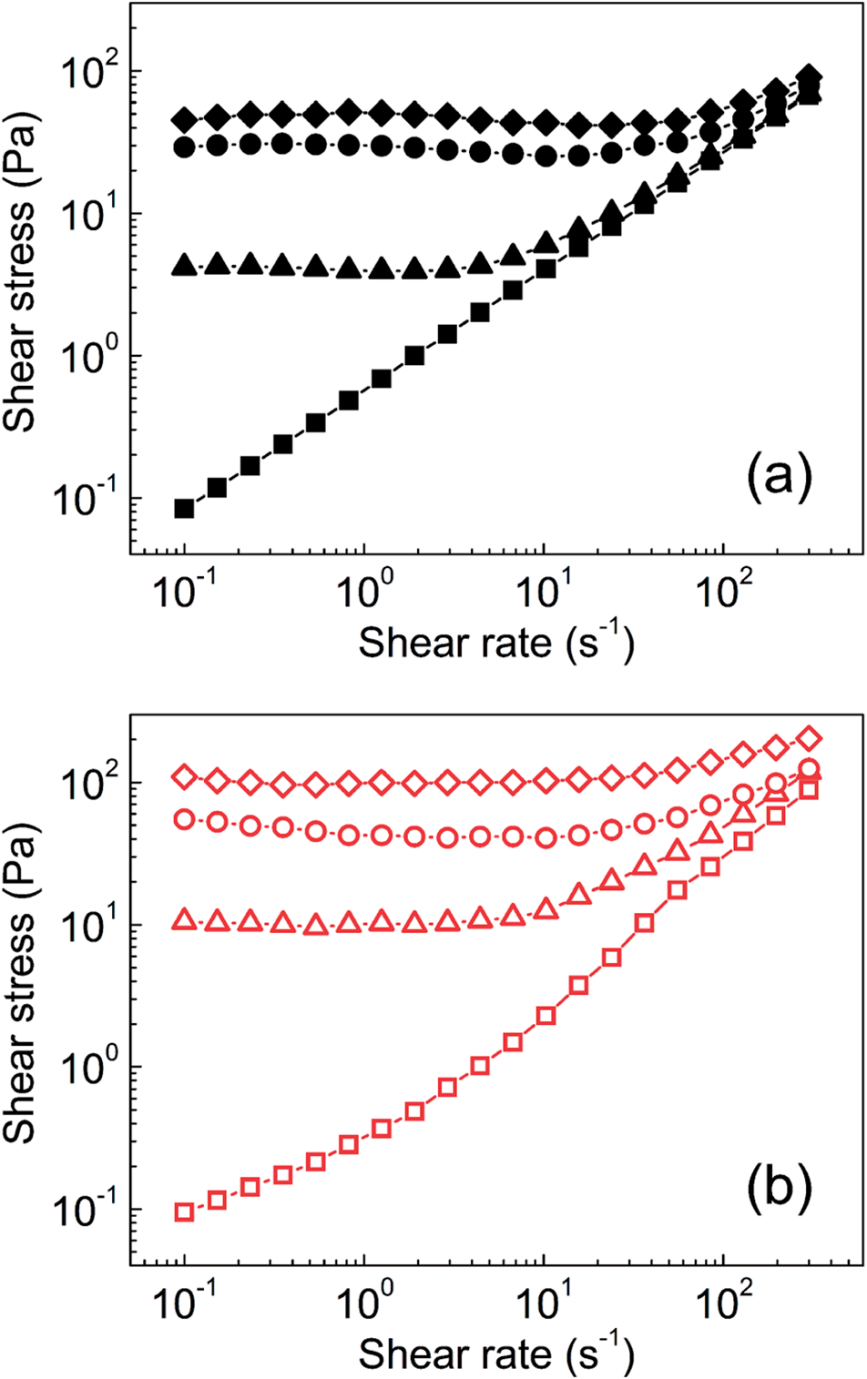
Steady shear flow curves of silicone oil suspensions for (a) neat GO (solid black symbols) and (b) GO-PBMA (open red symbols) at external electric field strengths of 0 (□, ■), 0.5 (△, ▲), 1.5 (○, ●), and 2.5 kV mm^−1^ (◇, ◆).

Steady shear rheological investigation is commonly used to quantify the ER capability of graphene oxide-based silicone oil suspensions.^18,20,31^ As shown in Fig. 6a, the neat GO-based suspension exhibited near-Newtonian behaviour in the absence of an external electric field. When an electric field of 0.5 kV mm^−1^ was switched on, the formation of partial chain-like structures was clearly visible (Fig. 5b) and the behaviour became pseudoplastic, as reflected by a yield stress of 4.6 Pa. Further increasing the external field to 1.5 kV mm^−1^ and 2.5 kV mm^−1^ resulted in increased yield stresses of 24 Pa and 41 Pa, respectively. In contrast, GO modified with PBMA had increased particle conductivity and better interactions with PDMS, resulting in the considerably different behaviour of this sample. The behaviour was also near-Newtonian in the absence of an external field, with a slight deviation resulting from the better interactions of GO-PBMA particles with silicone oil, similar to those already reported for GO-PGMA analogues.^46^ After application of the external electric field (0.5 kV mm^−1^) and formation of relatively strong chain-like structures (Fig. 5d), a yield stress of 10.5 Pa was achieved (Fig. 6b). Similar to previous reports, the yield stress further increased with increasing external electric field, reaching nearly 110 Pa at 2.5 kV mm^−1^, which was significantly higher than the value obtained for a common suspension based on GO, of approximately 35 Pa.^17^ Additional studies dealing with variously modified graphenes, GO, and newly reported materials and their ER performance are summarized in Table 2. Therefore, the results obtained from the GO-PBMA-based system were very promising because it contained only 5 wt% of particles in the suspension.

**Table 2:** State-of-the-art systems based on GO, graphene, and recently published TiO_2_/MoS_2_, and their ER performances at 3 kV mm^−1^

Sample name	Particle fraction in wt% or vol%	Shear stress [Pa]	Dielectric relaxation strength [—] and relaxation time [s]	Reference
Polyaniline/graphene	10 vol%	1300	N/A, N/A	47
Polypyrrole/reduced GO	5 vol%	600	N/A, N/A	48
Mesoporous silica/graphene	5 vol%	400	N/A, 3.2 × 10^−7^	49
Oligomeric silsesquioxane/GO	3 vol%	400	N/A, N/A	3
Flower-like TiO_2_ wrapped with MoS_2_	10 wt%	90	0.41, 7.4 × 10^−5^	50

**Fig. 7:**
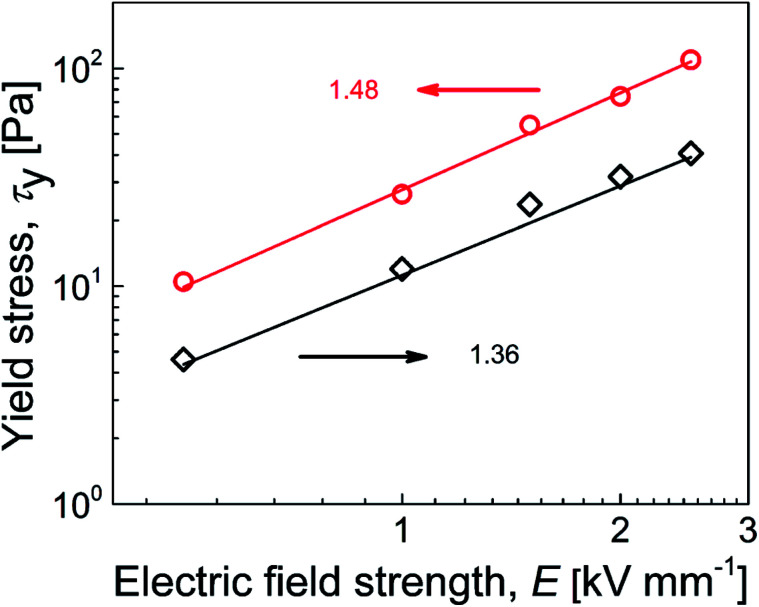
Dependence of yield stress on electric field strength for suspensions containing neat GO (◇) and GO-PBMA (○). Solid lines correspond to the power-law model fit.

To investigate the mechanisms of internal chain-like structure formation, the dependence of yield stress on the electric field strength was plotted and parameters of the power-law model fit (Table 3) were used to investigate this phenomenon, similar to previous reports.^51,52^ There are two mechanisms of internal structure formation. The first is the conductivity mechanism, when the coefficient *α* from eqn (1) reaches 1.5, which is based on the conductivity mismatch between the particles and silicone oil. The second mechanism is the polarization mechanism, when coefficient *α* reaches 2, which reflects the relative permittivity mismatch between semiconducting particles and silicone oil.^11,53,54^ For suspensions of neat GO, the value of *α* was found to be 1.36 (Fig. 7), indicating the conductivity mechanism. However, the value was considerably deviated from 1.5, indicating structures that were not precisely developed, in good agreement with the optical microscopy results (Fig. 5b). For the GO-PBMA-based suspension, parameter *α* was determined to be 1.48, which was in good agreement with conductivity mechanism and the well-developed internal chain-like structures (Fig. 5d). Furthermore, the *q* parameter, reflecting the rigidity of the chain-like structures, was found to be 11.2 and 27.7 for neat GO and GO-PBMA-based suspensions, respectively, which correlated well with results obtained from dielectric investigations described in a later section.

**Table 3:** Parameters of power-law model fits for neat and modified GO particle suspensions. Parameters *q* and *a* are properly described in the experimental section

Sample Code	*q* [Pa]	*a*
GO	11.2	1.36
GO-PBMA	27.7	1.48

### Dielectric properties

**Fig. 8:**
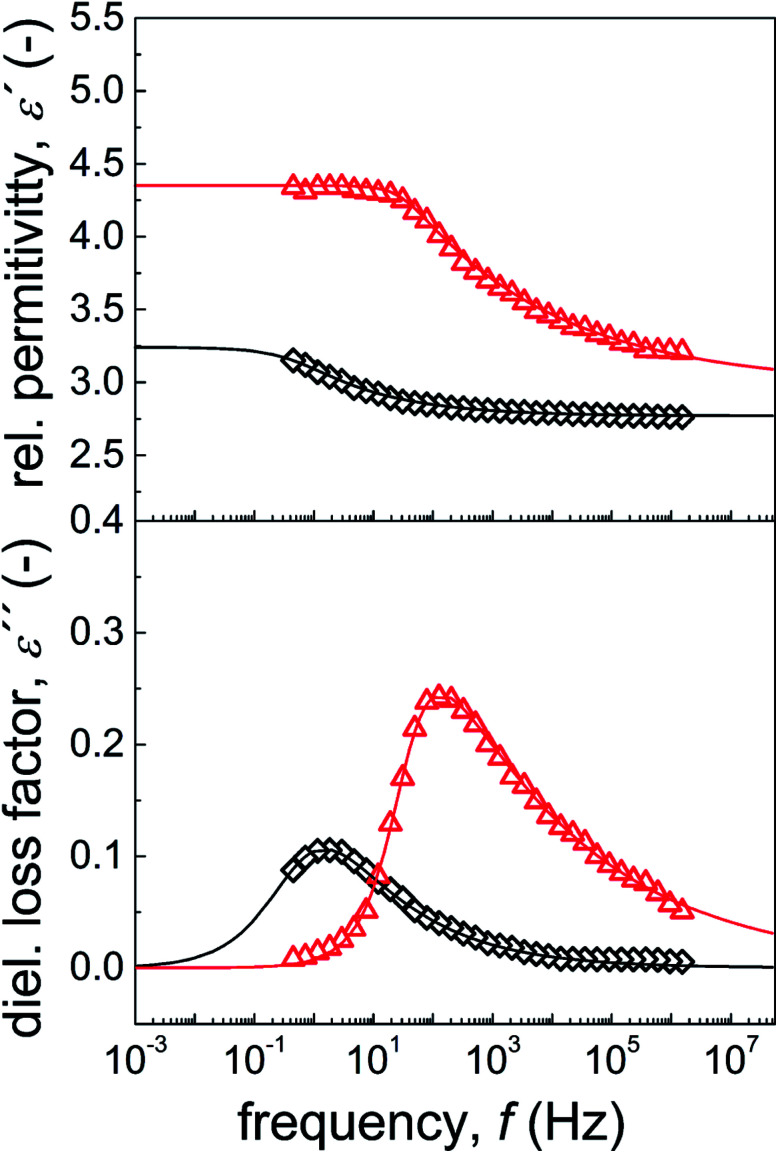
Frequency dependence of the relative permittivity and dielectric loss factor of neat GO (◇) and GO-PBMA (△) particles dispersed in silicone oil.

Dielectric properties are important characteristics for ER suspensions because there is a correlation between the ER performance and dielectric properties.^55^ Two crucial parameters, namely, dielectric relaxation strength (Δ*ε*′) and relaxation time (*t*_rel_), which can be obtained from the Havriliak–Negami model fit (eqn (2)), were shown to significantly influence the magnitude of ER performance. Generally, the ER performance increases with increasing Δ*ε*′ and decreasing *t*_rel_. Therefore, optimal dielectric properties were crucial for an ER system with enhanced ER effect. These values from neat GO-based suspensions were not appropriate, with Δ*ε*′ and *t*_rel_ values of 0.59 and 0.21 s, respectively (Fig. 8 and Table 4). In contrast, when GO was modified with PBMA, partial reduction of the GO and improved wettability with silicone oil (Fig. 8, inset image) resulted in improved dielectric characteristics, with Δ*ε*′ and *t*_rel_ values of 1.37 and 5 × 10^−3^ s, respectively (Fig. 8 and Table 4). Such significant improvements were well-correlated with the results obtained from both optical microscopy and electrorheological studies in the presence of an external field, confirming the enhanced ER performance of the GO-PBMA-based suspensions. Therefore, this system seems highly promising in comparison with other GO or layered systems, as summarized in Table 2.

**Table 4:** Parameters of Havriliak–Negami model for GO and GO-PBMA suspensions. Parameters are described in full in the Experimental section

Sample Code	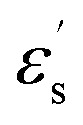	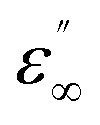	Δ*ε*′	*t*_rel_ [s]	*a*	*b*
GO	3.33	2.74	0.59	0.21	0.75	0.43
GO-PBMA	4.35	2.98	1.37	0.005	0.99	0.18

### Compatibility with silicone oil

**Fig. 9:**
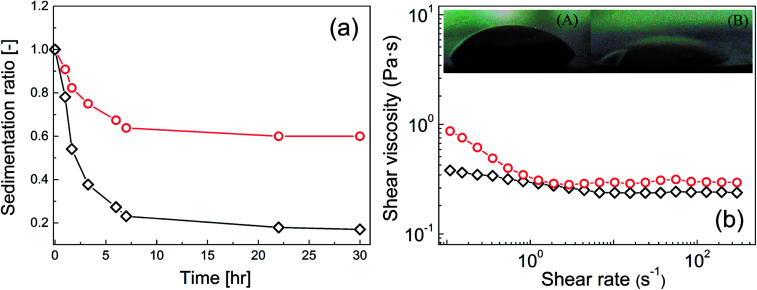
(a) Sedimentation ratio of neat GO (◇) and GO-PBMA (○) particles dispersed in silicone oil, and (b) steady shear viscosity dependence on the shear rate for neat GO (□) and GO-PBMA (△) particles in an external electric field strength of 0 kV mm^−1^ for the same suspensions. Insets represent the contact angle measurement for (A) neat GO and (B) GO-PBMA particles in the form of pellet using the sessile drop method.

The final part of this study focused on the compatibility of neat GO and GO-PBMA particles with silicone oil. This behaviour was crucial for the real applicability of these systems. Very weak compatibility of the dispersed phase with silicone oil would be expected to lead to significant particle sedimentation, because neat GO has a 2.5-times higher density than neat silicone oil and neat GO has poor wettability. Therefore, three experiments to determine the magnitude of compatibility between the particles and liquid medium were performed, namely, contact angle measurements, rheological investigation of the shear viscosity, and a sedimentation test. The neat GO particles showed a relatively high contact angle of 47.8 ± 2.9° (Fig. 9b, inset image (A)) due to rather small interactions of neat GO with the dispersed phase, and constant shear viscosity values at various shear rates (Fig. 9b). Finally, neat GO showed very poor sedimentation stability (Fig. 9a). In contrast, GO modified with PBMA chains showed significantly improved wettability with silicone oil. The contact angle decreased to 26.8 ± 2.3° (Fig. 9b, inset image (B)), reflecting enhanced interactions of the GO-PBMA particles with silicone oil and resulting in more pseudoplastic behaviour being observed (Fig. 9b). Consequently, improved sedimentation stability was also observed for GO-PBMA (Fig. 9a), with a sedimentation ratio three times higher than that of neat GO, while the particle density had only decreased to 2.21 g cm^−3^. Therefore, it could be stated that the improved sedimentation stability was mostly caused by improved compatibility, while a lower density probably only marginally contributed to this decrease.

## Conclusion

In this study, the modification of GO particles with PBMA chains using a SI-ATRP approach was performed to confirm that this technique was promising for the development of novel dispersed phases for ER systems. The polymerization of BMA was found to be well controlled regarding molar mass and dispersity. The compact coating of GO with PBMA was confirmed by TGA-FTIR, TEM, and AFM investigations. Targeted partial and simultaneous reduction of GO during SI-ATRP was confirmed by XPS analysis, the conductivity increased from 1 × 10^−8^ S cm^−1^ to 6 × 10^−7^ S cm^−1^, and the *I*_d_/*I*_g_ ratio increased from 0.90 to 1.09 in the Raman spectra. These findings were promising for applications in ER fluids, which were elucidated in detail using optical microscopy, steady shear rheology, and dielectric property measurements. Suspensions based on the GO-PBMA hybrid system exhibited well-developed internal chain-like structures with a yield stress of 110 Pa, dielectric relaxation strength of 1.37, and relaxation time of 5 × 10^−3^ s. Finally, the real-life applicability of this system was confirmed by the significantly enhanced sedimentation stability caused by enhanced interactions with silicone oil, as confirmed by the decrease in contact angle and shear viscosity profile. Therefore, the modification of GO with PBMA chains accompanied by partial GO reduction provided an ER system with enhanced sedimentation stability and improved ER properties compared with the system based on neat GO or previously described systems based on GO modified with acrylate-based polymers.

## Conflicts of interest

There are no conflicts to declare.

## Supplementary Material
